# PCR-Based Identification of Oral Streptococcal Species

**DOI:** 10.1155/2016/3465163

**Published:** 2016-09-14

**Authors:** Jeffrey A. Banas, Min Zhu, Deborah V. Dawson, Huojun Cao, Steven M. Levy

**Affiliations:** ^1^Iowa Institute for Oral Health Research, University of Iowa College of Dentistry, Iowa City, IA 52242, USA; ^2^Department of Preventive and Community Dentistry, University of Iowa College of Dentistry, Iowa City, IA 52242, USA

## Abstract

The microbial etiology of dental caries is still debated. Among the hypothesized contributors are the “low pH streptococci,” a designation given to unusually acid proficient strains among the primary plaque colonizers* S. oralis*,* S. mitis*,* S. gordonii*, and* S. anginosus*. However, accurate assignment of species is difficult among the oral streptococci. Our objective was to develop a streamlined method for identifying strains of* S. oralis* and* S. mitis* from plaque samples so that they could be analyzed in a separate study devoted to low pH streptococci and caries. Two independent PCR amplifications of a locus highly conserved among streptococci were used for presumptive species identification. Multilocus sequence analysis (MLSA) was used to measure accuracy. Sensitivity was 100% for selecting* S. oralis* and* S. mitis* among the clones sampled. Specificity was good except for the most closely related species that could not be reliably distinguished even by MLSA. The results with* S. oralis* and* S. mitis* were used to identify new primer sets that expanded the utility of the approach to other oral streptococcal species. These novel primer sets offer a convenient means of presumptive identification that will have utility in many studies where large scale, in-depth genomic analyses are not practical.

## 1. Introduction

The microbial etiology of dental caries has been the subject of numerous investigations that have spanned decades, if not centuries. The literature supports a prominent role for the mutans streptococci (MS) [1], especially in the earlier phases of caries development. However, there is still debate over the extent to which other plaque species influence the contribution of the MS, as well as general acknowledgment that there are pathways to caries that are independent of the MS [2–6]. In at least one instance, strain specificity rather than species specificity has been proposed [7–10]. The “low pH streptococci” is a designation given to unusually acid proficient strains of the non-MS oral species* Streptococcus mitis*,* Streptococcus oralis*,* Streptococcus gordonii*, and* Streptococcus anginosus*, though additional species have been suggested as well [9]. These atypical acid proficient variants are hypothesized to play a role in caries initiation, perhaps paving the way for elevations of the MS or other highly acidogenic species that foster disease progression.

Among contemporary investigations, the use of high throughput genetic means of species assignment is common. These studies have provided a valuable reexamination of the caries-related microbiome that is far more comprehensive than is practical with culture based studies. However, the genetic based studies are limited to correlating particular species with caries or health. Actual clones that can be analyzed for properties and characteristics that could explain a causal link generally are not obtained in these types of investigations. Additionally, there is no simple genetic basis for distinguishing the low pH streptococcal strains from less acid proficient strains among the relevant species.

In preparation for an investigation of low pH streptococci on incipient lesions, we sought to isolate strains of* S. oralis* and* S. mitis* for examination of acid properties. Speciation of viridans streptococci is complicated by both a high degree of genetic similarity between species and a relatively high level of genetic heterogeneity within species. We initially employed a traditional biochemical approach, based on reports in the literature [11–13], to identify low pH streptococci among isolates obtained after plating plaque samples on media selective for streptococci. The accuracy of the biochemical screening was low, particularly for identification of* S. mitis*, which some sources suggest is the most abundant streptococcal species within coronal dental plaque [13, 14]. In response, we developed a PCR screen that can be used to identify species associated with the low pH phenotype and validated the approach using multilocus sequence analysis (MLSA).

## 2. Materials and Methods

### 2.1. Isolation of Streptococcal Clones

Banked site-specific plaque samples (−80°C storage) from a long-term, ongoing study [18] were used in this investigation. They consisted of 170 samples from incipient decay or sound surfaces of permanent second molars in 85 subjects (38 male, 47 female; 79 White, 1 Black, 3 Hispanic, 1 Asian, and 1 Native American). Samples were retrieved from storage, thawed, vortexed, and diluted 1 : 1000 and 50 *μ*L was inoculated onto TYC medium agar plates [19] and onto Difco*™* Mitis-Salivarius agar (Becton, Dickinson and Co. Sparks, MD) for the selective growth of oral streptococci. Plates were incubated 48 hours in an aerobic atmosphere with 5% CO_2_ at 37°C.

### 2.2. Streptococcal Standard Strains

Standard strains were used for evaluating species specificity of the PCR amplifications and as controls in the bile solubility test. These strains were* S. oralis* ATCC 35037,* S. parasanguinis* ATCC 15912,* S. gordonii* ATCC 33399,* S. mitis* ATCC 49456,* S. constellatus* ATCC 27823,* S. mutans* ATCC 25175,* S. sanguinis* ATCC 10556,* S. intermedius* ATCC 27335,* S. sobrinus* ATCC 33478,* S. anginosus* ATCC 33397,* S. vestibularis* ATCC 49124,* S. salivarius* ATCC 25975,* S. cristatus* ATCC 51100,* S. infantis* ATCC 700779,* S. pseudopneumoniae* ATCC BAA-960, and* S. pneumoniae* ATCC 10813.

### 2.3. Polymerase Chain Reaction (PCR) Screen for Low pH Species

In order to select colonies from the TYC or Mitis-Salivarius plates in proportion to their presence in the plaque sample, all colonies (usually 24 to 28) from a distinct sector of the plate were picked and subcloned for future storage and isolation of DNA. DNA was isolated using the DirectAmp*™* Tissue Genomic DNA Amplification Kit (Denville Scientific Inc.; Holliston, MA). PCR reactions were set up for 25 *μ*L reactions using 2 *μ*L DNA template and reaction components from the GoTaq® Green Master Mix (Promega; Madison, WI). Reaction conditions were 95°C for 5 minutes, followed by 30 cycles of 95°C for 30 seconds, primer specific annealing temperature (see Table 1) for 30 seconds, and 72°C for 40 seconds. Reactions were programmed to conclude with 5 minutes at 72°C and a 4°C hold. Positive and negative reactions were determined by agarose (1%) gel electrophoresis and DNA visualization using SYBR Safe DNA gel stain (Invitrogen; Carlsbad, CA). Reactions were performed independently three times and gave identical results each time. Representative gels are shown in the figures. Primer sequences and annealing temperatures are shown in Table 1.

### 2.4. AP-PCR

All clones giving a PCR product with the GDH1 and/or GDH2 primer sets were evaluated for genotype identity using arbitrary-primed PCR. Primer 434 (5′-GCACAACAGTTCCCTGACTTGCAC-3′) [20] (1 *μ*L) was mixed with 4 *μ*L template DNA in a 20 *μ*L reaction using the GoTaq Green Master Mix. Reaction conditions were 95°C for 2 minutes, followed by 45 cycles of 94°C for 1 minute, 30°C for 1 minute, and 72°C for 2 minutes. Reactions were programmed to conclude with 5 minutes at 72°C and a 16°C hold. Amplification patterns were observed following agarose (2%) gel electrophoresis at 70 V for 1.5 hours. A strain was designated a unique amplitype if any portion of its DNA banding pattern was nonidentical to other strains isolated from the same tooth site.

### 2.5. Multilocus Sequence Analysis (MLSA)

Randomly selected strains were analyzed by MLSA to assign and/or confirm speciation. Gene selection, primer sequences, and PCR reaction conditions were taken from Bishop et al. [21] for* map*,* pfl*,* ppaC*,* pyk*,* rpoB*,* sodA*, and* tuf*. PCR products were purified using the QIAquick PCR Purification Kit (QIAGEN; Hilden, Germany) and sent for DNA sequencing to the University of Iowa Genomics Division within the Iowa Institute of Human Genetics. Results were deposited with the National Center for Biotechnology Information as LIBGSS_039316. Since the internet application described in [21] was under repair during the time of this study, gene sequences were evaluated for species identity using the NCBI BLAST program and the microbial database.

To create a dendrogram of relatedness of the presumptive* S. oralis* and* S. mitis* clones that were analyzed by MLSA, sequence was used from four (*map*,* pfl*,* ppaC*, and* tuf*) of the seven genes that gave the most consistent results of high Sanger sequencing quality. For each of the genes at least 300 bp were included. The concatenated sequences were aligned using the Clustal W algorithm [22]. The phylogenetic inference was based on the Maximum Likelihood Method using MEGA7 [16]. The reliability of each tree topology was checked by 500 bootstrap replications.

### 2.6. Lysis by Sodium Deoxycholate

In order to distinguish* S. pneumoniae* from closely related* S. mitis* and* S. pseudopneumoniae* species, a bile solubility test was performed. In this test, suspensions of* S. pneumoniae* become clear in the presence of 2% sodium deoxycholate due to cell lysis, whereas nonpneumococcal species are resistant or only partially lysed. Briefly, test organisms and standard strains were grown overnight on 5% sheep's blood agar aerobically with 5% CO_2_ at 37°C. Growth from the blood agar plate was used to make a turbid suspension of the organism (OD_600_ = 0.5 to 1.0) in 0.85% saline, which was then divided equally into two tubes. An equal volume of 2% sodium deoxycholate was added to one tube and an equal volume of 0.85% saline was added to the second tube. The tubes were mixed, incubated at 37°C, and examined every 10 minutes up to 2 hours for clearing of turbidity in the sodium deoxycholate tube relative to the saline control.

## 3. Results and Discussion

**Figure 1:**
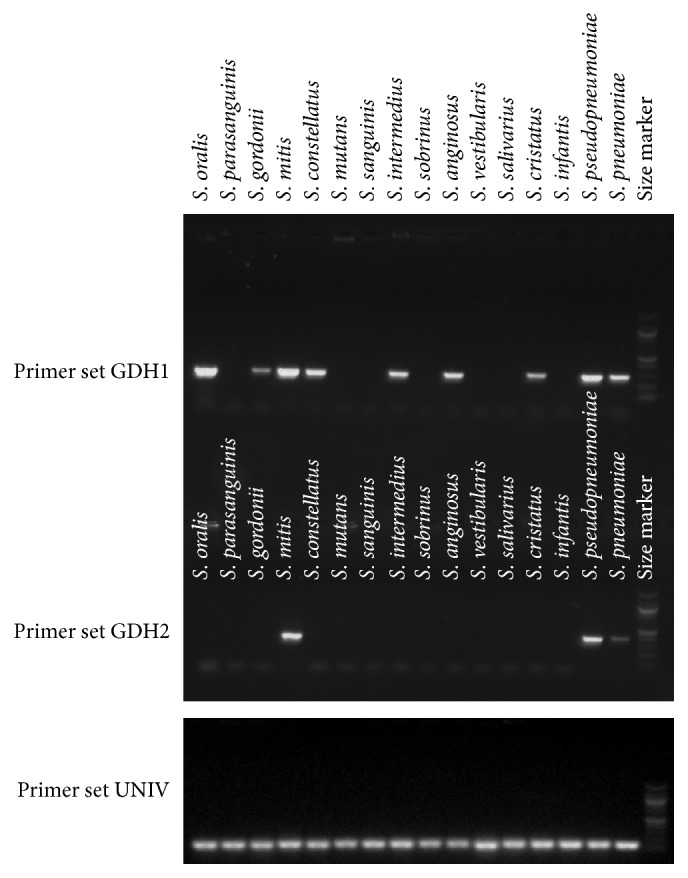
Primer sets GDH1 and GDH2 were evaluated for specificity by testing for amplification of a PCR product from DNA isolated from a panel of ATCC type strains representing species typically found in the oral cavity. Representative gels are shown. The DNA preps used for all primer set amplifications were confirmed to yield product with a set of universal primers (UNIV) for the 16S rRNA gene.

Streptococcal genome comparisons using the NCBI BLAST tool led to the identification of a highly conserved gene, encoding glucose-3-phosphate 1-dehydrogenase (*gdh*), which contained a variable region that offered the possibility of species-specific primer sites together with intraspecies conservation. Three primers were synthesized to form two primer sets that shared the same forward primer but utilized unique reverse primer sequences. The first primer set (designated GDH1) was predicted to amplify a product from a subset of low pH streptococci,* S. mitis* and* S. oralis*, whereas the second primer set (designated GDH2) was predicted to amplify a product only from* S. mitis*. We focused on* S. oralis* and* S. mitis* because these species were predicted to be the most highly represented among the putative low pH species [13]. The specificity of the primer sets was predicted to be excellent but not absolute. DNA from most other plaque streptococcal species was not expected to yield a product, with the notable exceptions of* S. pneumoniae* and* S. pseudopneumoniae*.* S. pneumoniae* is not likely to be a major presence in dental plaque, but there is little information regarding the prevalence of* S. pseudopneumoniae*. Using type strains from the American Type Culture Collection (ATCC), we tested the specificity of primer sets GDH1 and GDH2. Both primer sets amplified products from the predicted species, although the GDH1 primers gave products from some species that had not been predicted. Fortuitously, products were obtained from each of the putative low pH species, as well as additional species of* Streptococcus* (*S. constellatus*,* S. cristatus*) that have been statistically linked to dental caries [4, 23, 24] (Figure 1).

The primer sets then were used to screen 4080 streptococcal clones isolated from the plaque samples described in Materials and Methods. From this screen 623 GDH1+/GDH2− (hereafter designated GDH1+) clones and 226 GDH1+/GDH2+ (hereafter designated GDH2+) clones were identified. AP-PCR results determined that there were 324 unique amplitypes among the GDH1+ isolates and 138 unique amplitypes among the GDH2+ isolates. To verify species identity, MLSA was performed on 10% of the unique GDH1+ and 10% of GDH2+ amplitypes, both of which were chosen by a random number generator. In addition, 20 random GDH1−/GDH2− (hereafter designated GDH0) clones were analyzed by MLSA to determine the sensitivity of the GDH1 and GDH2 primer sets. The results, shown in Table 2, reveal that the majority of GDH1+ isolates were most similar to an unnamed strain designated VT162 [25].* S. oralis* was the closest named species for most of the GDH1+ isolates. All of the GDH2 isolates were most similar to* S. mitis*,* S. pseudopneumoniae*, or* S. pneumoniae* (see Table S1 in Supplementary Material available online at http://dx.doi.org/10.1155/2016/3465163 for full MLSA results). However,* S. pneumoniae* was ruled out by a test for lysis by sodium deoxycholate (Figure S1). Additionally, the standard strain of* S. pseudopneumoniae* grew more slowly on TYC medium than blood agar, leaving open the possibility that this species was underrepresented in our initial isolation and that our GDH2+ strains are most likely* S. mitis*. GDH0 isolates most often were* S. mutans*, along with* S. gordonii*,* S. sanguinis*, and* S. salivarius*. Thus,* S. gordonii* strains may or may not yield a product with the GDH1 primer set. This observation could be consistent with the relatively weak product amplification seen with GDH1+* S. gordonii* isolates (Figure 1) and variability in genetic relatedness among* S. gordonii* strains [21]. Attempts to adjust the specificity of the primer sets by raising the annealing temperature abruptly eliminated amplification from all species except* S. mitis*.

The MLSA of GDH1+ strains most often had variable species assignment, depending on the gene sequence used (Table S1). There was excellent agreement from gene to gene for milleri streptococcal species* S. intermedius* and* S. anginosus*. Surprisingly, two GDH1+ strains appeared to be* S. sanguinis* even though the standard* S. sanguinis* strain did not yield a PCR product with the GDH1 primer set. Gene-specific species assignments varied between* S. oralis* and strain VT162 for all 24 strains that were assigned as VT162 or VT162/*S. oralis*. Strain VT162 is reported to be most closely related to* S. oralis*, but with an average nucleotide identity of less than 95%, suggesting the possibility of a novel species [25]. For GDH1+ and GDH2+ strains, the* sodA* and* pyk* gene homology scores often were the lowest among the gene panel. Nonetheless, the MLSA confirmed that all of the GDH2+ strains were most closely related to* S. mitis*,* S. pseudopneumoniae*, and* S. pneumoniae*, as predicted. For GDH0 strains, the* sodA* gene failed to give a product for a majority of isolates. No* S. mutans* strains yielded a product for the* map* gene. The gene panel was based on the work of Bishop et al. [21] and gave satisfactory results, though in our hands a substitute for the* sodA* gene could be warranted.

**Figure 2:**
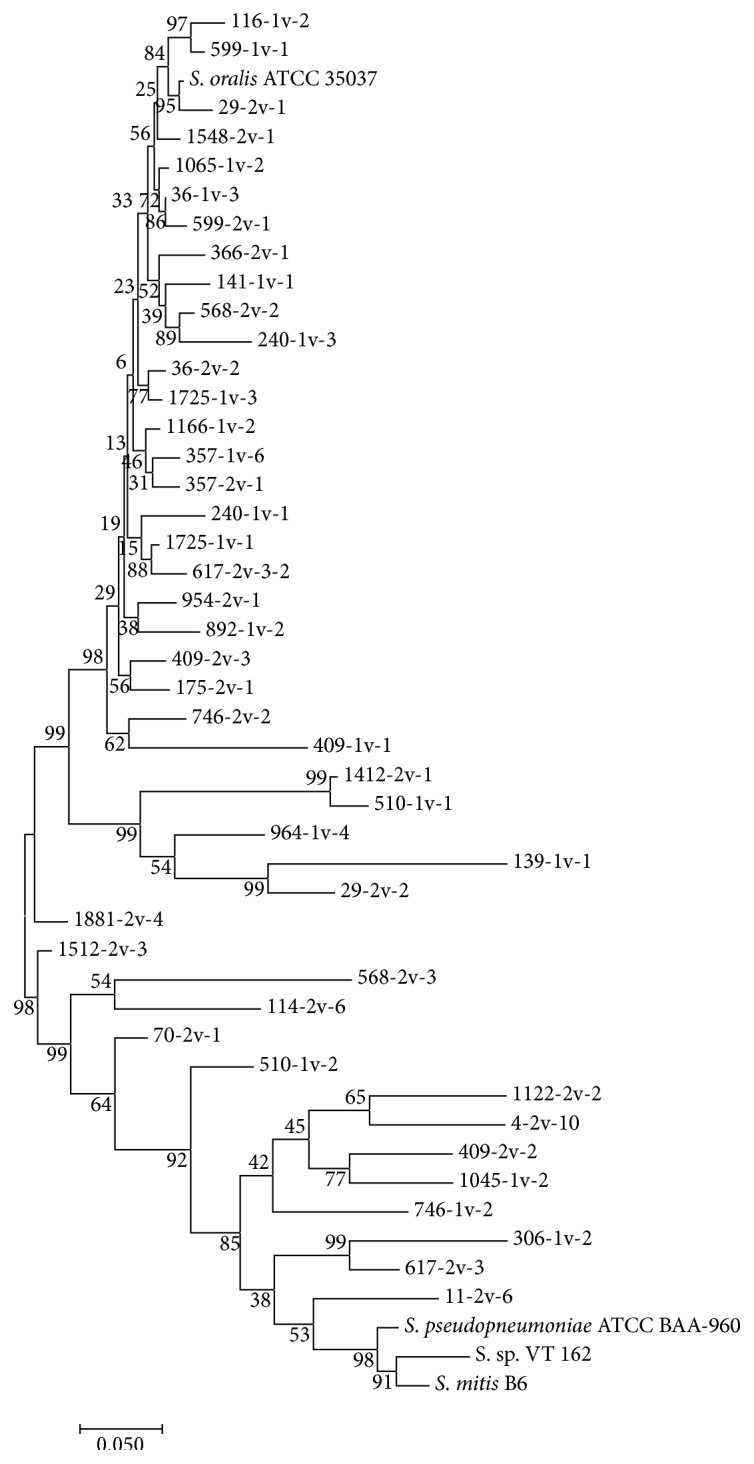
Molecular phylogenetic tree of MLSA concatenated sequences for the map, pfl, ppaC, and tuf genes. The evolutionary history was inferred by using the Maximum Likelihood Method based on the Tamura-Nei model [15]. The tree with the highest log likelihood is shown. The percentage of trees in which the associated taxa clustered together is shown next to the branches. Initial tree(s) for the heuristic search were obtained automatically by applying Neighbor-Join and BioNJ algorithms to a matrix of pairwise distances estimated using the Maximum Composite Likelihood (MCL) approach and then selecting the topology with superior log likelihood value. The tree is drawn to scale, with branch lengths measured in the number of substitutions per site. Evolutionary analyses were conducted in MEGA7 [16]. The prospective* S. oralis* isolates (upper portion of the dendrogram from 116-1v-2 to 29-2v-2) appear segregated from the prospective* S. mitis* isolates (1881-2v-4 to 11-2v-6). Among the prospective* S. oralis* isolates, 1412-2v-1 (S*. intermedius*), 510-1v-1 (*S. intermedius*), 964-1v-4 (uncertain species assignment), 139-1v-1 (*S. gordonii*), and 29-2v-2 (*S. sanguinis*) were assigned to species other than* S. oralis* and map furthest from the* S. oralis* control. Two prospective* S. oralis* strains (96-2v-4 and 1881-2v-3) were not included due to poor sequence quality in the regions of alignment.

Four genes,* map*,* pfl*,* ppaC*, and* tuf*, were selected on the basis of consistent Sanger sequencing quality to generate a dendrogram of strain relatedness for GDH1+ and GDH2+ isolates compared to GenBank sequence data for* S. oralis*,* S. mitis*,* S. pseudopneumoniae*, and VT162 controls (Figure 2). While the VT162 control surprisingly segregated with the* S. mitis* isolates and control rather than the* S. oralis* isolates, presumptive* S. oralis* isolates were most closely related to the* S. oralis* control and the presumptive* S. mitis* isolates were most closely related to* S. mitis* and* S. pseudopneumoniae* controls as expected and corroborating the effectiveness of the GDH1/2 primer approach.

**Figure 3:**
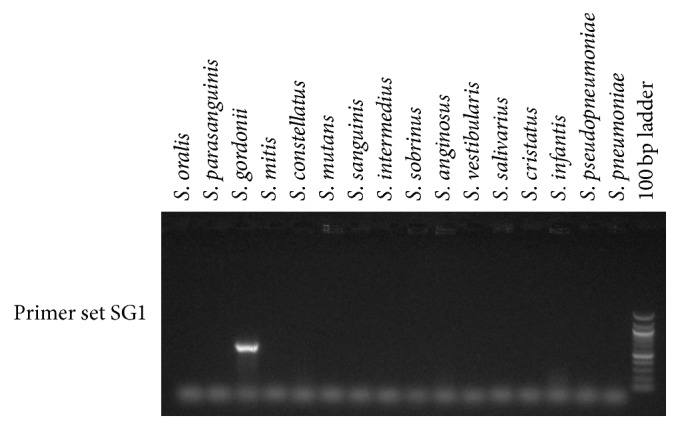
Primer set SG1 was evaluated for specificity by testing for amplification of a PCR product from DNA isolated from a panel of ATCC type strains representing species typically found in the oral cavity. A representative gel is shown.

**Figure 4:**
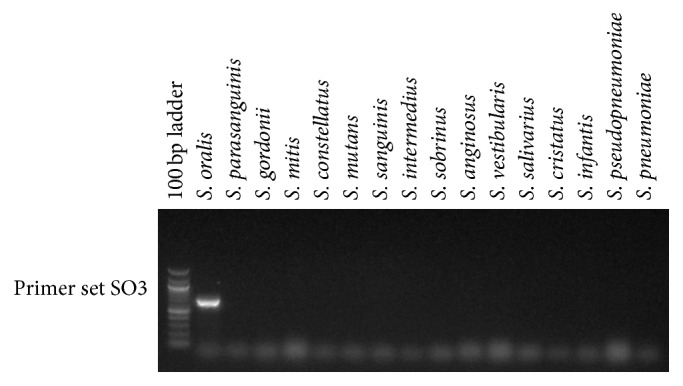
Primer set SO1 was evaluated for specificity by testing for amplification of a PCR product from DNA isolated from a panel of ATCC type strains representing species typically found in the oral cavity. A representative gel is shown.

**Figure 5:**
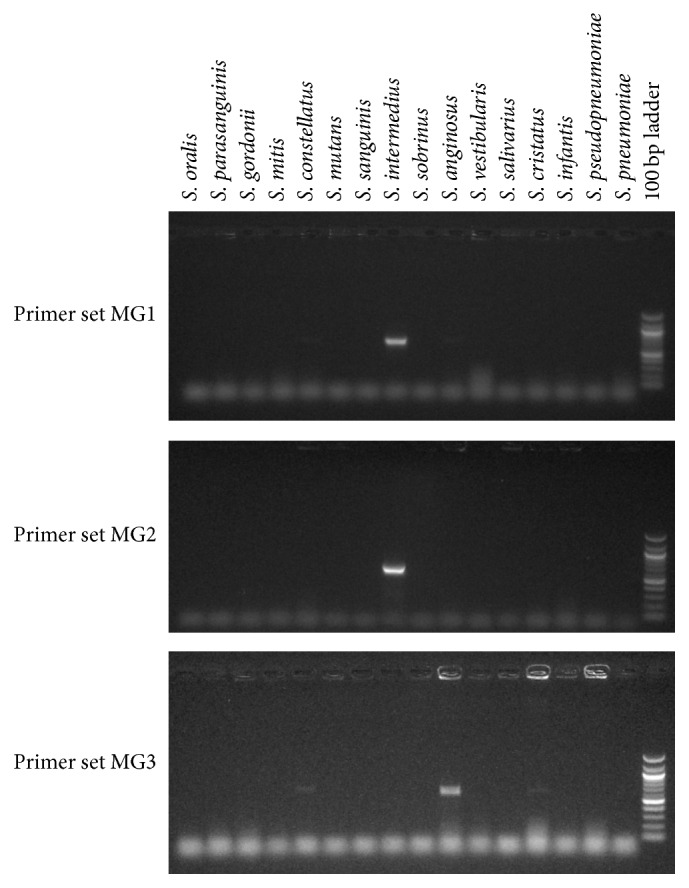
Primer sets MG1, MG2, and MG3 were evaluated for specificity by testing for amplification of a PCR product from DNA isolated from a panel of ATCC type strains representing species typically found in the oral cavity. Representative gels are shown.

While the GDH1 amplification was very good overall in identifying the streptococcal species linked to low pH strains (*S. oralis*,* S. mitis*,* S. gordonii*, and* S. anginosus*), it lacked some sensitivity in picking up* S. gordonii* and lacked some specificity with respect to milleri streptococci. To overcome these limitations, we explored whether different primer pairs from the same* gdh* gene locus could better identify* S. gordonii* and distinguish among members of the milleri streptococci. When tested on the GDH1+ and GDH0 MLSA strains, it was found that primer set SG1 amplified a product only from* S. gordonii* among the standard strains (Figure 3), from all the GDH0* S. gordonii* strains (data not shown) and from the 1 GDH1+* S. gordonii* strain (data not shown). Thus, the SG1 primer set will be useful for screening for* S. gordonii* strains that might otherwise be missed by the GDH1 primer set. Further refining specificity for* S. oralis* among GDH1+ strains was accomplished with primer set SO3 (Figure 4). For distinguishing the milleri streptococci, three primer sets, MG1, MG2, and MG3, were tested (Figure 5). MG1 and MG2 amplified bright bands from* S. intermedius* DNA, whereas* S. anginosus* and* S. constellatus* DNA only resulted in weak bands from MG1. MG3 amplified a strong band from* S. anginosus* DNA and yielded weak bands from DNA of* S. constellatus* and* S. cristatus*.

Other potential species-specific primer sets have been identified based on unique amplification using the panel of ATCC standard strains and could ultimately be useful in future studies of low pH streptococci. These include primer sets for* S. sanguinis*,* S. parasanguinis*, and* S. infantis*. Future studies would also benefit from being able to distinguish strains of* S. cristatus*. While a primer set specific for this species has not yet been identified, we have found a primer set that yields strong amplification products from only* S. cristatus* and* S. constellatus*. When coupled with the MG primer sets, it is possible to differentiate* S. cristatus*.

The importance of obtaining physical isolates of putative low pH streptococci was reinforced by comparisons of colony morphology among the strains analyzed by MLSA. Two observations stood out. First, colony morphologies were identical or highly similar within the GDH1+ and GDH2+ groups when grown on the streptococcal selection medium TYC but showed much greater variability on 5% sheep's blood agar. Second, as a consequence, blood agar could be used to test the uniformity of colony characteristics among strains that appeared to share the same species identity based upon the genetic analyses. It was easy to distinguish milleri group streptococci from* S. oralis*/VT162 among the GDH1+ strains, and there was a maximum of two colony morphologies among the 25 strains designated as VT162 by MLSA. In contrast, there were at least seven different colony morphologies on blood agar among the 14 GDH2+ strains identified as* S. mitis*/*pseudopneumoniae*/*pneumoniae* by MLSA. Strikingly, none of the seven morphologies matched the characteristics of the ATCC standard strains for these species. These observations highlight not only the complexity and difficulty in distinguishing among the viridans streptococci, but also the limitations of large scale studies that assign speciation based solely on the DNA sequences of 16S rRNA genes. This is evident particularly among putative* S. mitis* [26], a species that could exhibit unusually high levels of genetic variability and/or enjoy apparent numerical dominance due to misidentification and close relatedness to several different species, some of which are unrecognized.

It might be suggested that isolation of low pH streptococcal strains can simply be done on low pH media and that there is no need for a genetic based protocol. Indeed, this is the means by which some low pH variants were initially recognized [9]. Nonetheless, this straightforward approach has its limitations. First, there is the implicit assumption that acidogenic potential will parallel the acid tolerant property of growing on low pH media. While plausible, there is no specific information for how well these acid properties correlate for various streptococcal species. Our preliminary work with the set of standard strains indicates a lack of correlation. Second, low pH medium isolation must be done in conjunction with neutral pH isolation if one is to determine the relative frequency of finding low pH variants belonging to a particular species. These are not trivial considerations as they could help explain, along with differences and limitations inherent in 16S rDNA probes, why some species, for example,* S. cristatus*, have been found to correlate both with caries [23] and with health [27, 28].

## 4. Conclusions

Accurate speciation of the viridans streptococci presents an enormous challenge even for state-of-the-art genetic comparisons. Expensive, labor-intensive approaches are not practical for high throughput studies. We began with a goal of picking out strains of* S. oralis* and* S. mitis* from among total oral streptococci. In order to do so we developed a PCR-based means of presumptive identification that bested enzymatic screens. We further developed the method to expand its utility to other oral streptococcal species. Sensitivity is excellent and specificity is sufficient to distinguish all but the most closely related species. While we employed this PCR-based tool in the context of investigating caries etiology, its utility should extend to a variety of investigations that require a method of screening for presumptive identities among the oral streptococci, including studies related to oral ecology, health, and disease.

## Supplementary Material

The supplementary table provides additional data for each strain tested by MLSA. The highest homology matches (species plus score) for each of the seven genetic loci used in the MLSA are provided.

## Figures and Tables

**Table 1:** Primer sequences and annealing temperatures.

Primer set	Forward (5′ to 3′)	Reverse (5′ to 3′)	Product size (bp)	Annealing temperature (°C)
GDH1	ACAACTGAAACCTTTGCATCTGG	CGGTCGCATCTGTACGGTAA	278	51
GDH2	Same as GDH1	TCAAYTTCCAYGAYGCACCA	391	51
SG1	CCTTGGAGCAAGGAATATTTTGAATCTG	CTTCTTGGCTCGGTTGTGTCAAGCG	695	53
SO3	Same as SG1	AGAGCGATATTGACCACGAATAAAC	732	53
MG1	Same as SG1	CCCGTCTACTTTACCAGAAGTA	715	51
MG2	Same as SG1	GTCTTTTCAGATCATTATCAGTAGGC	706	51
MG3	CGTGGTCGAGTCTATCACAGATCTCGCT	CAGAAACAAAGGTTTCAGTGGTAGAC	788	53
UNIV^*∗*^	GTGSTGCAYGGYTGTCGTCA	ACGTCRTCCMCACCTTCCTC	~200	60

^*∗*^Positive control primers, universal for bacterial 16S rRNA, were chosen based on [17].

**Table 2:** Species assignment based on MLSA.

Strains	Number tested	Species distribution
GDH1+	32	25 *Streptococcus* sp. VT162/*S. oralis* 1 *S. gordonii* 2 *S. intermedius* 1 *S. anginosus* 2 *S. sanguinis* 1 Uncertain species affiliation

GDH2+	14	13 *S. mitis*/*pseudopneumoniae*/*pneumoniae* 1 Uncertain species affiliation

GDH0	20	9 *S. mutans* 3 *S. sanguinis* 3 *S. gordonii* 2 *S. salivarius* 3 Undetermined; no PCR products

## References

[1] Tanzer J. M., Livingston J., Thompson A. M. (2001). The microbiology of primary dental caries in humans. *Journal of Dental Education*.

[2] Gross E. L., Beall C. J., Kutsch S. R., Firestone N. D., Leys E. J., Griffen A. L. (2012). Beyond *Streptococcus mutans*: dental caries onset linked to multiple species by 16S rRNA community analysis. *PLoS ONE*.

[3] Aas J. A., Griffen A. L., Dardis S. R. (2008). Bacteria of dental caries in primary and permanent teeth in children and young adults. *Journal of Clinical Microbiology*.

[4] Simón-Soro A., Belda-Ferre P., Cabrera-Rubio R., Alcaraz L. D., Mira A. (2013). A tissue-dependent hypothesis of dental caries. *Caries Research*.

[5] Kleinberg I. (2002). A mixed-bacteria ecological approach to understanding the role of the oral bacteria in dental caries causation: an alternative to *Streptococcus mutans* and the specific-plaque hypothesis. *Critical Reviews in Oral Biology and Medicine*.

[6] Marsh P. D. (1994). Microbial ecology of dental plaque and its significance in health and disease. *Advances in Dental Research*.

[7] van Houte J. (1994). Role of micro-organisms in caries etiology. *Journal of Dental Research*.

[8] Nyvad B., Kilian M. (1990). Comparison of the initial streptococcal microflora on dental enamel in caries-active and in caries-inactive individuals. *Caries Research*.

[9] Svensäter G., Borgström M., Bowden G. H. W., Edwardsson S. (2003). The acid-tolerant microbiota associated with plaque from initial caries and healthy tooth surfaces. *Caries Research*.

[10] van Ruyven F. O. J., Lingström P., van Houfe J., Kent R. (2000). Relationship among mutans streptococci, ‘low-ph’ bacteria, and lodophilic polysaccharide-producing bacteria in dental plaque and early enamel caries in humans. *Journal of Dental Research*.

[11] Beighton D., Hardie J. M., Whiley R. A. (1991). A scheme for the identification of viridans streptococci. *Journal of Medical Microbiology*.

[12] Facklam R. (2002). What happened to the streptococci: overview of taxonomic and nomenclature changes. *Clinical Microbiology Reviews*.

[13] Tappuni A. R., Challacombe S. J. (1993). Distribution and isolation frequency of eight streptococcal species in saliva from predentate and dentate children and adults. *Journal of Dental Research*.

[14] Simón-Soro Á., Tomás I., Cabrera-Rubio R., Catalan M. D., Nyvad B., Mira A. (2013). Microbial geography of the oral cavity. *Journal of Dental Research*.

[15] Tamura K., Nei M. (1993). Estimation of the number of nucleotide substitutions in the control region of mitochondrial DNA in humans and chimpanzees. *Molecular Biology and Evolution*.

[16] Kumar S., Stecher G., Tamura K. (2016). MEGA7: Molecular Evolutionary Genetics Analysis version 7.0 for bigger datasets. *Molecular Biology and Evolution*.

[17] Filkins L. M., Hampton T. H., Gifford A. H. (2012). Prevalence of streptococci and increased polymicrobial diversity associated with cystic fibrosis patient stability. *Journal of Bacteriology*.

[18] Broffitt B., Levy S. M., Warren J., Cavanaugh J. E. (2013). Factors associated with surface-level caries incidence in children aged 9 to 13: the Iowa Fluoride Study. *Journal of Public Health Dentistry*.

[19] De Stoppelaar J. D., Van Houte J., De Moor C. E. (1967). The presence of dextran-forming bacteria, resembling *Streptococcus bovis* and *Streptococcus sanguis*, in human dental plaque. *Archives of Oral Biology*.

[20] Rudney J. D., Larson C. J. (1999). Identification of oral mitis group streptococci by arbitrarily primed polymerase chain reaction. *Oral Microbiology and Immunology*.

[21] Bishop C. J., Aanensen D. M., Jordan G. E., Kilian M., Hanage W. P., Spratt B. G. (2009). Assigning strains to bacterial species via the internet. *BMC Biology*.

[22] Larkin M. A., Blackshields G., Brown N. P. (2007). Clustal W and Clustal X version 2.0. *Bioinformatics*.

[23] Tanner A. C. R., Mathney J. M. J., Kent R. L. (2011). Cultivable anaerobic microbiota of severe early childhood caries. *Journal of Clinical Microbiology*.

[24] Becker M. R., Paster B. J., Leys E. J. (2002). Molecular analysis of bacterial species associated with childhood caries. *Journal of Clinical Microbiology*.

[25] Vecherkovskaya M. F., Tetz G. V., Tetz V. V. (2014). Complete genome sequence of the *Streptococcus* sp. strain VT 162, isolated from the saliva of pediatric oncohematology patients. *Genome Announcements*.

[26] Kemp M., Bangsborg J., Kjerulf A. (2013). Advantages and limitations of ribosomal RNA PCR and DNA sequencing for identification of bacteria in cardiac valves of Danish patients. *Open Microbiology Journal*.

[27] Preza D., Olsen I., Aas J. A., Willumsen T., Grinde B., Paster B. J. (2008). Bacterial profiles of root caries in elderly patients. *Journal of Clinical Microbiology*.

[28] Kanasi E., Dewhirst F. E., Chalmers N. I. (2010). Clonal analysis of the microbiota of severe early childhood caries. *Caries Research*.

