# Supplementary material for: PCR-Based Identification of Oral Streptococcal Species
Source: Int J Dent. 2016 Sep 14;2016:3465163. doi: 10.1155/2016/3465163 (PMC5039290; doi:10.1155/2016/3465163)
Supplement: Supplementary file 1 — The supplementary table provides additional data for each strain tested by MLSA. The highest homology matches (species plus score) for each of the seven genetic loci used in the MLSA are provided. [file 3465163.f1.pdf]

TABLE S1 Highest Homology Scores for Each Gene of MLSA

GDH1+	*map*	*pfl*	*ppaC*	*pyk*	*ropB*	*sodA*	*tuf*	Species Assignment
1	VT162 (457)	VT162 (540)	VT162 (834)	VT162 (343)	Oralis (774)	I-P16 (138)	VT162 (654)	Strep sp. VT162
2	Gordonii (297)	Gordonii (408)	Gordonii (796)	Mitis (468)	Gordonii (618)	Gordonii (316)	Gordonii (609)	*S. gordonii*
3	VT162 (547)	VT162 (533)	VT162 (823)	VT162 (508)	Mitis (755)	VT162 (284)	VT162/Oralis (646)	Strep sp. VT162
4	Oralis (520)	VT162 (522)	VT162 (812)	VT162 (444)	VT162 (778)	Oralis (475)	VT162 (655)	Strep sp. VT162
5	VT162 (522)	VT162 (545)	VT162 (780)	VT162 (187)	Oralis (774)	Oralis (79)	VT162 (590)	Strep sp. VT162
6	Gordonii (482)	Gordonii (524)	Gordonii (783)	< (50)	Gordonii (765)	Gordonii (394)	Gordonii (260)	*S. gordonii*
7	Oralis/VT162 (462)	VT162 (489)	VT162 (785)	VT162 (484)	VT162 (756)	VT162 (95)	VT162 (661)	Strep sp. VT162
8	Oralis/VT162 (466)	Mitis (499)	VT162 (785)	Mitis/VT162 (125)	VT162 (740)	I-62/I-P16 (141)	VT162 (621)	Strep sp. VT162
9	Oralis (453)	VT162 (482)	VT162 (810)	VT162 (581)	VT162 (773)	Oralis (354)	VT162 (646)	Strep sp. VT162
10	VT162 (219)	VT162 (522)	VT162 (787)	VT162 (572)	Oralis (769)	VT162 (154)	VT162 (636)	Strep sp. VT162
11	Oralis/VT162 (482)	VT162 (511)	VT162 (845)	VT162 (605)	Oralis (747)	VT162 (331)	Oralis (668)	Strep sp. VT162
12	Oralis/VT162 (489)	VT162 (491)	VT162 (812)	VT162 (601)	Oralis (747)	Oralis (443)	Oralis (664)	*S. oralis*/VT162
13	VT162 (544)	VT162 (401)	Oralis (832)	VT162 (641)	Oralis (147)	Mitis (123)	VT162 (675)	Strep sp. VT162
14	VT162/Oralis (401)	VT162 (513)	Oralis (215)	VT162/Mitis (565)	VT162 (159)	Oralis (86)	VT162 (590)	Strep sp. VT162
15	Oralis (464)	Mitis (489)	VT162 (805)	VT162 (563)	VT162/Oralis (416)	Pneumoniae (113)	VT162 (616)	Strep sp. VT162
16	Intermedius (544)	Intermedius (538)	Intermedius (792)	Intermedius (542)	Intermedius (623)	Intermedius (156)	Intermedius (655)	*S. intermedius*
17	VT162 (475)	VT162 (509)	VT162 (755)	VT162 (668)	Pseudopn./Oralis (255)	VT162 (340)	VT162 (663)	Strep sp. VT162
18	VT162 (508)	VT162 (491)	VT162 (780)	VT162 (749)	Oralis (82)	Oralis (291)	VT162 (654)	Strep sp. VT162

19	VT162 (520)	VT162 (504)	VT162 (827)	VT162 (603)	VT162 (325)	Oralis (79)	VT162/Oralis (627)	Strep sp. VT162
20	Oralis (479)	VT162 (482)	Oralis (789)	VT162 (684)	VT162 (241)	Oralis (241)	VT162 (592)	Strep sp. VT162
21	Oralis (338)	VT162 (495)	Oralis (818)	< (50)	VT162 (107)	VT162 (100)	VT162 (609)	Strep sp. VT162
22	VT162/Oralis (500)	VT162 (535)	VT162 (769)	VT162 (187)	< (50)	< (50)	Oralis (522)	Strep sp. VT162
23	VT162 (479)	VT162/Oralis (515)	VT162 (767)	Mitis (122)	VT162 (639)	Oralis (190)	VT162 (569)	Strep sp. VT162
24	Anginosus (313)	Mitis (489)	Pseudopneumo (594)	< (50)	VT162 (145)	Mitis (149)	Parasanguinis (551)	Uncertain
25	Anginosus (544)	Anginosus (535)	VT162 (178)	Anginosus (648)	Anginosus (812)	Anginosus (533)	Anginosus (670)	*S. anginosus*
26	VT162/Oralis (506)	VT162 (549)	VT162 (830)	VT162 (331)	VT162 (783)	Oralis (432)	VT162 (664)	Strep sp. VT162
27	VT162/Oralis (493)	VT162 (535)	VT162 (818)	VT162 (645)	< (50)	Oralis (497)	Oralis (677)	Strep sp. VT162
28	Intermedius (542)	Intermedius (551)	Intermedius (960)	Intermedius (563)	Intermedius (818)	Intermedius (452)	Intermedius (681)	*S. intermedius*
29	Oralis (524)	VT162 (540)	VT162 (794)	VT162 (594)	Oralis (774)	Mitis (277)	VT162 (652)	Strep sp. VT162
30	Oralis (488)	VT162/Oralis (526)	Oralis (794)	VT162 (589)	VT162 (765)	VT162 (291)	VT162 (659)	Strep sp. VT162
31	VT162 (502)	VT162 (549)	VT162 (843)	VT162 (600)	VT162/Oralis (731)	VT162 (347)	VT162 (675)	Strep sp. VT162
32	Oralis (389)	Gordonii (459)	Mitis (493)	Gordonii (695)	Gordonii (650)	No Product	Gordonii (636)	*S. gordonii*
GDH2+	*map*	*pfl*	*ppaC*	*pyk*	*ropB*	*sodA*	*tuf*	Species Assignment
1	Pseudopneumo (109)	Pneumoniae (488)	Pneumoniae (223)	< (50)	Mitis (558)	Mitis (214)	Pseudopneumo (648)	Mitis/Pseudo/Pneumo
2	Mitis (140)	Pneumoniae (210)	Mitis (800)	Mitis (298)	Mitis (569)	Pneumoniae (302)	Pseudopneumo (583)	*S. mitis*
3	Mitis (253)	Pneumoniae (515)	Pneumoniae (733)	Mitis (280)	Mitis (295)	Pneumoniae (304)	Pseudopneumo (659)	*S. mitis/pneumoniae*
4	Mitis (455)	Pneumoniae (450)	Mitis (360)	Pseudopn./Pneumo (100)	Pseudopn./Pneumo (228)	Pneumoniae (132)	Pseudopneumo (522)	Mitis/Pseudo/Pneumo

5	Mitis (123)	Pseudopn./Pneumo (390)	Pseudopneumo (336)	Gordonii (95)	Mitis (576)	I-G2/I-P16 (138)	Pseudopn./Mitis (520)	*S. pseudopn./mitis*
6	Pseudopneumo (109)	Pseudopneumo (471)	Pneumoniae (475)	< (50)	Pseudopneumo (369)	< (50)	Pseudopn./Mitis (639)	*S. pseudopneumoniae*
7	Pseudopneumo (170)	Mitis (482)	Mitis (713)	Mitis (239)	Mitis (178)	Mitis (107)	Pseudopnuemo (641)	*S. mitis*
8	< (50)	Pseudopn./Pneumo (484)	Mitis (464)	Pseudopneumo (136)	Mitis (159)	VT162/Mitis (79)	Mitis (637)	*S. mitis*
9	Pneumoniae (149)	Pseudopneumo (403)	Pseudopneumo (585)	< (50)	Pseudopneumo (255)	Pneumoniae (145)	Pseudopneumo (623)	*S. pseudopneumoniae*
10	Mitis (105)	Pseudopneumo (443)	Mitis (513)	< (50)	Pseudopn./Pneumo (349)	< (50)	Mitis (583)	*S. mitis*
11	Mitis (185)	Pneumoniae (336)	Pneumoniae (540)	< (50)	Pneumoniae (320)	Mitis (141)	Mitis (639)	*S. mitis/pneumoniae*
12	Pneumoniae (389)	Pseudopneumo (428)	Pneumoniae (396)	Mitis (372)	Mitis (661)	Pneumoniae (174)	Mitis (661)	*S. mitis/pneumoniae*
13	Mitis (477)	Pneumoniae (527)	Pneumo/Mitis (499)	Mitis (672)	Pseudopn./Mitis (100)	Mitis (445)	Pseudopneumo (535)	*S. mitis*
14	Mitis (482)	Pneumoniae (518)	Mitis (857)	Mitis (569)	Pseudopneumo (769)	Mitis (407)	VT162 (621)	*S. mitis*

GDH0	*map*	*pfl*	*ppaC*	*pyk*	*ropB*	*sodA*	*tuf*	Species Assignment
1	Gordonii (517)	Gordonii (493)	Gordonii (960)	Intermedius (55)	Gordonii (711)	Gordonii (300)	Gordonii (679)	*S. gordonii*
2	No Product	Mutans (437)	Mutans (960)	Mutans (257)	Mutans (816)	No Product	Mutans (681)	*S. mutans*
3	No Product	Mutans (430)	Mutans (966)	Mutans (417)	Mutans (778)	No Product	Mutans (681)	*S. mutans*
4	Sanguinis (540)	Sanguinis (372)	Sanguinis (940)	< (50)	Sanguinis (800)	No Product	Sanguinis (684)	*S. sanguinis*
5	No Product	No Product	No Product	No Product	No Product	No Product	No Product	Indeterminate
6	Sanguinis (466)	Sanguinis (385)	Sanguinis (839)	VT162 (50)	Gordonii (690)	Sanguinis (345)	Sanguinis (661)	*S. sanguinis*
7	No Product	No Product	No Product	No Product	No Product	No Product	No Product	Indeterminate
8	Sanguinis (489)	Sanguinis (378)	Sanguinis (832)	Sanguinis (581)	Sanguinis (688)	No Product	Sanguinis (673)	*S. sanguinis*
9	No Product	Mutans	Mutans	< (50)	Mutans	No Product	Mutans	*S. mutans*

		(208)	(960)		(805)		(681)	
10	No Product	Mutans (435)	Mutans (966)	Mutans (677)	Sanguinis (710)	No Product	Mutans (690)	*S. mutans*
11	Gordonii (471)	Gordonii (533)	Gordonii (801)	Gordonii (590)	Gordonii (782)	Gordonii (508)	Gordonii (636)	*S. gordonii*
12	Salivarius (522)	Salivariuis (482)	No Product	Salivarius (693)	No Product	Salivarius (480)	Salivarius (668)	*S. salivarius*
13	Gordonii (468)	Gordonii (464)	Gordonii (834)	Gordonii (506)	Gordonii (783)	Gordonii (535)	Gordonii (654)	*S. gordonii*
14	No Product	No Product	No Product	No Product	No Product	No Product	No Product	Indeterminate
15	Salivarius (509)	Salivarius (495)	Salivarius (852)	Salivarius (183)	Salivarius (791)	Salivarius (461)	Salivarius (677)	*S. salivarius*
16	No Product	Mutans (430)	Mutans (827)	Mutans (176)	Mutans (812)	No Product	Mutans (681)	*S. mutans*
17	No Product	Mutans (230)	Mutans (955)	Mutans (464)	Mutans (825)	No Product	Mutans (690)	*S. mutans*
18	No Product	Mutans (464)	Mutans (419)	Mutans (695)	Mutans (823)	No Product	Mutans (690)	*S. mutans*
19	No Product	Mutans (517)	Mutans (960)	Mutans (682)	Mutans (816)	No Product	Mutans (690)	*S. mutans*
20	No Product	Mutans (405)	Mutans (807)	Mutans (666)	Mutans (823)	No Product	Mutans (681)	*S. mutans*

Supplementary Figure 1

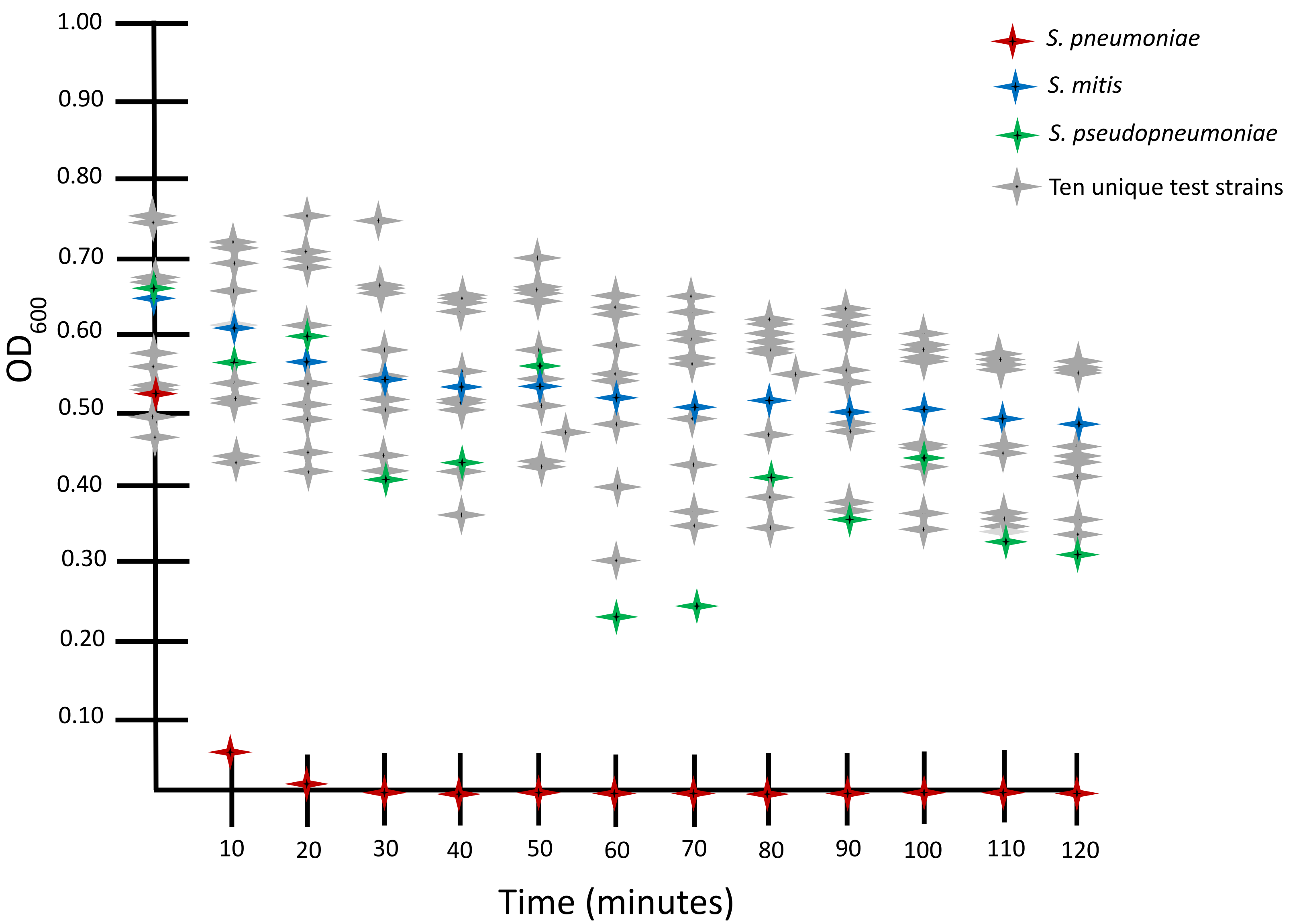

Putative *S. mitis* strains that showed strong homology with *S. pneumoniae* or *S. pseudopneumoniae* via MLSA, were tested for lysis by sodium deoxycholate. Only *S. pneumoniae* should lyse and exhibit an optical density (OD) near 0. A control strain of *S. pneumoniae*

showed complete lysis by 20 minutes whereas control strains of *S. mitis* and *S. pseudopneumoniae*, along with 10 suspected *S. mitis* strains, showed only partial or negligible lysis.
